# Non-Radioactive Detection of Trinucleotide Repeat Size Variability

**DOI:** 10.1371/currents.md.ad50113b899fa1352ce70c087eead706

**Published:** 2014-03-06

**Authors:** Stéphanie Tomé, Annie Nicole, Mario Gomes-Pereira, Genevieve Gourdon

**Affiliations:** INSERM UMR 1163, Laboratory of CTG repeat instability and myotonic dystrophy Paris Descartes – Sorbonne Paris Cité University, Imagine Institute, Paris, France; INSERM UMR 1163, Laboratory of CTG repeat instability and myotonic dystrophy Paris Descartes – Sorbonne Paris Cité University, Imagine Institute, Paris, France; INSERM UMR 1163, Laboratory of CTG repeat instability and myotonic dystrophy Paris Descartes – Sorbonne Paris Cité University, Imagine Institute, Paris, France; INSERM UMR 1163, Laboratory of CTG repeat instability and myotonic dystrophy Paris Descartes – Sorbonne Paris Cité University, Imagine Institute, Paris, France

## Abstract

Many human diseases are associated with the abnormal expansion of unstable trinucleotide repeat sequences. The mechanisms of trinucleotide repeat size mutation have not been fully dissected, and their understanding must be grounded on the detailed analysis of repeat size distributions in human tissues and animal models. Small-pool PCR (SP-PCR) is a robust, highly sensitive and efficient PCR-based approach to assess the levels of repeat size variation, providing both quantitative and qualitative data. The method relies on the amplification of a very low number of DNA molecules, through sucessive dilution of a stock genomic DNA solution. Radioactive Southern blot hybridization is sensitive enough to detect SP-PCR products derived from single template molecules, separated by agarose gel electrophoresis and transferred onto DNA membranes. We describe a variation of the detection method that uses digoxigenin-labelled locked nucleic acid probes. This protocol keeps the sensitivity of the original method, while eliminating the health risks associated with the manipulation of radiolabelled probes, and the burden associated with their regulation, manipulation and waste disposal.

## Introduction

The expansion of trinucleotide repeat sequences (TNRs) was identified as the causative mutation in more than 20 neurological and neuromuscular disorders, such as Huntington’s disease (HD), myotonic dystrophy type 1 (DM1), fragile X syndrome (FRAXA) and Friedreich’s ataxia (FRDA) 1. Non-affected individuals usually carry <40 repeats. Short repeats are stable in somatic tissues and stably transmitted from one generation to the next. Expanded, disease-associated repeats are usually longer than ~50 repeats, and highly unstable. Repeat instability is commonly expansion-biased, with a pronounced tendency to further increase in size in the germline and in the soma. Larger repeats are often associated with an earlier age of onset and a worsening of clinical severity, a process called anticipation. In the soma, TNRs instability is age-dependent, biased towards expansions, highly tissue-specific and is is probably associated with the progression of symptoms 2. Although mouse and cell culture models have provided significant insight into the mechanisms of size mutation, the dynamics of trinucleotide repeats instability have not been fully elucidated. Extensive analysis of repeat dynamics over time and across generations is essential for a complete understanding of the molecular mechanisms that generate repeat size diversity. Understanding the mechanisms of repeat size mutation will hopefully lead to novel therapeutic means to avoid repeat expansion, while promoting repeat contraction down to non-pathogenic lengths 3.

Standard methods for assessing trinucleotide repeat length variability are based on Southern blot analysis of restriction digestion or bulk PCR amplification of genomic DNA. These methods analyze simultaneously the expansion size in thousands of cells, underestimating rare mutant molecules in the complex population. Sensitive SP-PCR methods overcome this difficulty, through the serial dilution of bulk genomic DNA into multiple PCR amplifications, which resolve the heterogeneous smears, detected by standard Southern blot hybridization, into discrete amplification products derived from single cells 4. The detection of SP-PCR products generated by the amplification of a low number of input template DNA molecules required the use of Southern blot and sensitive hybridization techniques, using ^32^P-radiolabeled oligonucleotide probes 5. However, the use of radioelements of high emission energy in life sciences represents a real health risk and implies a heavy administrative paper work. To eliminate the health and safety risks, and to facilitate the implementation of this technique by laboratories and institutes, we have developed a non-radioactive method to detect repeat-containing SP-PCR products transferred onto a nylon membrane.

We have focused on the CTG repeat expansion analysis in DM1 patients and using a transgenic mouse model of DM1 6 ^,^ 7. To this end, we have used a digoxigenin(DIG)-labelled locked nucleic acid (LNA) oligonucleotide probe, previously designed for genomic DNA Southern blot hybridization 8. The protocol we describe here can be adapted to the detection of other trinucleotide repeat expansions, such as CAG repeats in HD and other polyglutamine expansion disorders, CGG repeats in FRAXA or GAA repeats in FRDA. We anticipate it to be also useful for the analysis of other unstable DNA sequences (e.g. pentanucleotide and hexanucleotide repeats). The technique is suitable for both human and mouse samples. When performed as we describe, the detection method is fast, reliable and sensitive enough to detect rare repeat sizes, providing important clues to the dissection of fundamental aspects of trinucleotide repeat biology.

## Materials

**A- Reagents**

-Digoxigenin-labeled DNA molecular Weight Marker III-Fragment sizes: 0.125 - 21.2 kb (21226, 5148, 4973, 4268, 3530, 2027, 1904, 1584, 1375, 947, 831, 564, 125 bp; Roche, cat. 11218603910)

-Digoxigenin-labeled DNA molecular Weight Marker VI-Fragment sizes: 0.15 - 2.1 kb (2176, 1766, 1230, 1033, 653, 517, 453, 394, 298, 234, 220, 154 bp); Roche, cat. 11218611910)

-Blocking reagent (Roche, cat. 11096176001)

-DIG antibody (Roche, cat. 11093274910)

-Ready-to-use CDP-Star (Roche, cat. 12041677001)

-DIG labelled LNA (Locked Nucleic acid) probe 8: 5DigN/GC^+^AG^+^CAGC^+^AG^+^CAGC^+^AGCA (Eurogentec, Scale 250nmole DNA and purification to HLPC; C^+^ means LNA cytosine and G^+^ means LNA guanine). Stock solution: 0.1nmol/µl.

-N-Lauroylsarcosine sodium salt (M=293,4, Sigma, L5125)

-20% SDS

-20X SSC

-Maleic acid (M=116.1, Sigma, M-0375)

-NaCl (M=58.44, Carlo Erba, 479687)

-Tris base (M=121.14, Applichem A2264)

-Tween 20

-NaOH

**B-Buffers**

**Maleic acid buffer 1x (0.1M maleic acid and 0.15M NaCl, pH: 7.5 with NaOH)**

11.61g maleic acid

8.72g NaCl

Qsp H_2_0 1000mL

Adjust pH to 7.5 with NaOH****

**10% Blocking buffer**

Add 10g of blocking reagent in 100mL of Maleic acid buffer. Heat in the microwave for less than 2 minutes to dissolve it. Avoid boiling it before shaking. Autoclave it.

**Hybridization buffer (5xSSC, 0.1% N-laurosyl sarcosine, 0.02% SDS, 1% blocking solution)**

125mL 20X SSC

0.05g Lauroylsarcosine sodium salt

500µl of 20% SDS

50mL of 10% blocking solution

Qsp H_2_O 500mL

**Low stringency buffer (2X SSC and 0.1% SDS)**

100mL of 20X SSC

5mL of 20% SDS

895mL H_2_O

**Medium Stringency (0.5X SSC and 0.1% SDS)**

25mL of 20X SSC

5mL of 20% SDS

970mL H_2_O

**Washing buffer (0.1M maleic acid, 0.15M NaCl and 0.3% Tween 20, pH 7.5 with NaOH)**

11.61g maleic acid

8.72g NaCl

3mL tween 20

Qsp H_2_0 1000mL

Adjust pH to 7.5 with NaOH

**Detection buffer (0.1M Tris and 0.1M NaCl, pH 9.5 with NaOH)**

6.05g Tris

2.9g NaCl

Qsp H_2_O 500mL

Adjust pH to 9.5 with NaOH

**C- Equipments**

- UV crosslinker (UV Stratalinker 2400, Stratagene, catalogue #400076 (230 V)

- Hybridization bottles of 200mL

- Temperature-controlled, rotating hybridization oven

- Heating block

- Tweezers

- Shaker

- Flat trays

- Stationary plastic sleeves

- Autoradiography cassettes

- X-ray film

- X-ray film processor

- Plasticware (1.5 mL plastic tubes, 50mL plastic tubes, pipette tips)

## Procedures

**A: **Following agarose gel electrophoresis of PCR products, **transfer DNA** onto a GeneScreen Plus® Hybridization Transfer Membrane by Southern squash blotting, as previously described 5 ****

**B: Fix the DNA** onto the membrane using a UV crosslinker and by exposure of each side of the membrane to 1200 J/m^2^ of UV radiation. Avoid drying the membrane.****

**C: Pre-Hybridization:**

-Add 20mL of hybridization buffer into a hybridization bottle, insert membrane with DNA side facing the inside of the bottle and taking care to avoid air bubbles. Air bubbles trapped must be removed by rolling a wet 10 mL pipette over the membrane to force them out of the edge of the blot.

-Incubate at 70°C for 30min with rotation.

**D: Hybridization:**

-Denature DIG-labeled LNA probe at 94° for 5 min, (2µl in 50µl hybridization buffer). Cool down the LNA probe on ice.

-Add the 52ul of LNA probe to fresh 20mL of hybridization buffer (final probe concentration: 10pmol/mL) and replace the hybridization buffer used for pre-hybridization by the new buffer supplemented with DIG-labeled probe

-Incubate at 70°C for 3h with rotation.

-Recover the hybridization buffer and probe following incubation into a 50 mL plastic tube and freeze it at -20°C. The LNA probe can be re-used at least 3 times, following denaturation in boiling water for 5 min and cooling down on ice.

**E: Post- Hybridization (with shaking):**

-Incubate membrane twice in ~200mL of low stringency buffer at RT for 5min in a flat tray with gentle shaking. The volume of buffer will depend on the size of used tray but it should be enough to fully cover the membrane. Avoid drying any portion of the membrane during any of the post-hybridization and detection steps.

-Incubate membrane three times in ~200mL of medium stringency buffer (preheated at 70°C) for 15 min at RT, with continuous shaking, keeping the membrane humidified throughout the entire procedure.

**F: Washing & blocking membrane**

-Transfer membrane onto a flat tray containing ~200mL of washing buffer and incubate at RT for 2min with gentle shaking.

-Discard washing buffer and transfer the DNA membrane into a hybridization bottle (DNA facing the inside).

-Dilute the 10% blocking solution with maleic acid buffer to obtain a 1% blocking solution. Add 25mL of the diluted 1% blocking solution in the hybridization bottle and incubate for 30 min at RT with rotation (do not replace the blocking solution by milk).

**G: Detection of PCR products with anti-DIG antibody**

-Add 1.25µl anti-DIG antibody to 25mL of fresh 1% blocking solution, to obtain a 1:20,000 antibody working dilution. Replace the 1% blocking solution by anti-DIG antibody diluted in 25ml of working dilution, and incubate for 30min at RT in the hybridization bottle with rotation.

-Transfer membrane into a flat tray containing ~200mL of washing buffer and wash twice for 15min at RT.

-Discard the washing buffer.

-Equilibrate the membrane in ~100 ml of detection buffer for 3 min at RT on a flat tray. Volume of detection buffer is approximate and it should be enough to fully cover the membrane, keeping it humidified throughout the entire procedure.

**H: Chemiluminescent detection**

-Place membrane on a plastic sleeve (open and flattened), with DNA facing up.

-Apply 1mL (20-30 drops) of Ready-to-use CDP-Star for every 100 cm2 of membrane. Apply solution dropwise over the entire surface of the blot until evenly distributed, to obtain a membrane that is homogenously soaked.

-Close plastic sleeve, covering the membrane and taking care to avoid air bubbles. Incubate membrane for 5 min at RT.

-Remove excess liquid and place the membrane (inside the plastic sleeve, DNA facing up), in an autoradiography cassette.

-Develop autoradiograph following an exposure time of 1-5 minutes. Examples of typical autoradiograph are presented in Figures 1 and 2.

**Detection of CTG repeat instability in blood from a 27 year-old DM1 patient carrying more than 170 CTG repeat:**
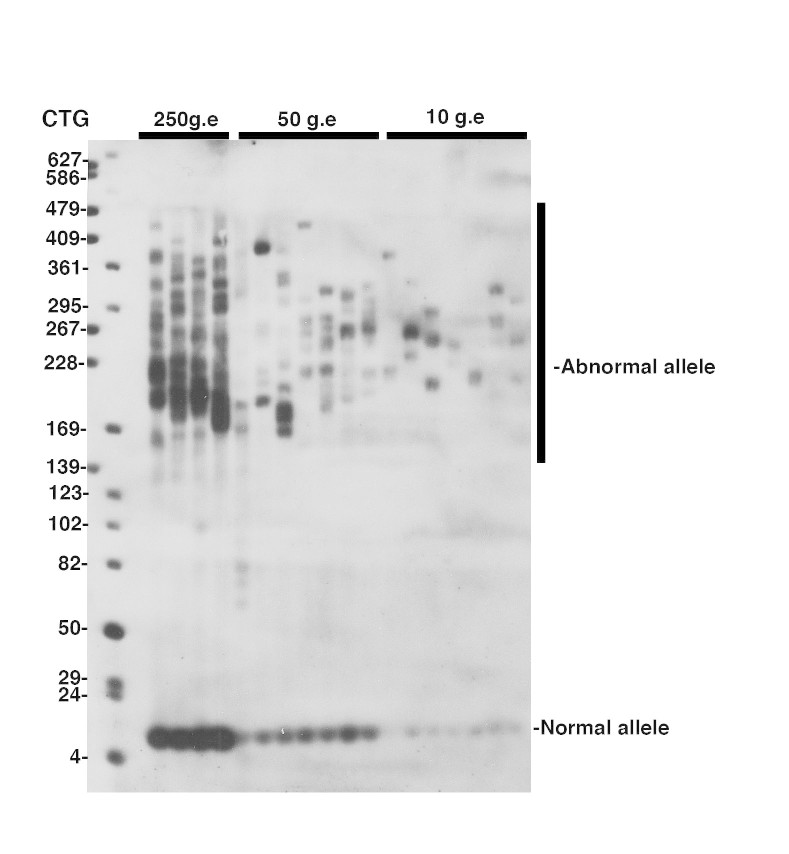
SP-PCR was performed as described by Gomes-Pereira et* al*, 2004. An average of 250, 50 and 10 genome equivalents (g.e.) were amplified in each replicate reaction, as indicated above the lanes. The membrane was hybridized with a non-radioactive CAG oligonucleotide probe. The medical RX autoradiography film was exposed for 5 minutes at room temperature.

**Detection of CTG repeat length in tail DNA from DM1 mice carrying a large fragment of human genomic DNA, comprising 45kb of the DM1 locus and more than 1200 of CTG repeats in the mouse genome (Gomes-Pereira et al, 2007).**
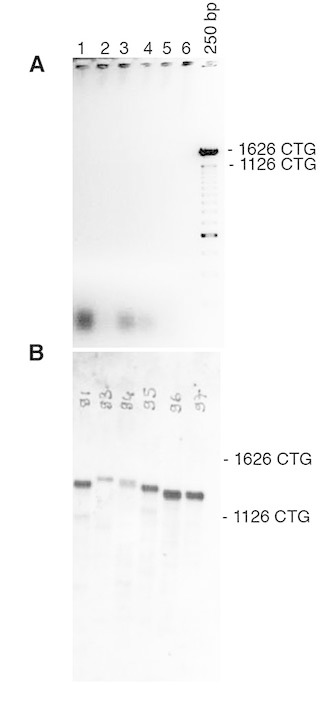
To amplify CTG repeat, bulk PCR was performed on 10-100ng of template genomic DNA, as previously described (Seznec et *al*, 2000) A: 25ul of PCR product were electrophoresed through a 0.7% agarose gel. Only faint or no signal was observed following ethidium bromide staining. B: The agarose gel was blotted and the PCR products detected by non-radioactive hybridization with DIG-labelled CAG probe. Our new detection method enables to reveal specific PCR products (stronger bands), not observed in A. The weaker and smaller bands represent non-specific PCR products, resulting from DNA polymerase slippage during bulk PCR amplification of large amounts of genomic DNA. Such PCR artifacts are rarely generated when amplified small amounts of template DNA, as in Figure 1

## Competing Interests

The authors have declared that no competing interests exist.
